# Mouse *N*-acetyltransferase type 2, the homologue of human *N*-acetyltransferase type 1

**DOI:** 10.1016/j.bcp.2007.12.012

**Published:** 2008-04-01

**Authors:** Akane Kawamura, Isaac Westwood, Larissa Wakefield, Hilary Long, Naixia Zhang, Kylie Walters, Christina Redfield, Edith Sim

**Affiliations:** a Department of Pharmacology, University of Oxford, Mansfield Road, Oxford OX1 3QT, United Kingdom; b Department of Biochemistry, Molecular Biology and Biophysics, University of Minnesota, Minneapolis, MN 55455, USA; c Department of Biochemistry, University of Oxford, South Parks Road, Oxford OX1 3QU, United Kingdom

**Keywords:** Arylamine *N*-acetyltransferase/NAT, Enzyme inhibition, Selective estrogen receptor modulator/SERM, Steroid, Xenobiotic, Breast cancer

## Abstract

There is increasing evidence that human arylamine *N*-acetyltransferase type 1 (NAT1, EC 2.3.1.5), although first identified as a homologue of a drug-metabolising enzyme, appears to be a marker in human oestrogen receptor positive breast cancer. Mouse Nat2 is the mouse equivalent of human NAT1. The development of mouse models of breast cancer is important, and it is essential to explore the biological role of mouse Nat2. We have therefore produced mouse Nat2 as a recombinant protein and have investigated its substrate specificity profile in comparison with human NAT1. In addition, we have tested the effects of inhibitors on mouse Nat2, including compounds which are endogenous and exogenous steroids. We show that tamoxifen, genistein and diethylstilbestrol inhibit mouse Nat2. The steroid analogue, bisphenol A, also inhibits mouse Nat2 enzymic activity and is shown by NMR spectroscopy, through shifts in proton peaks, to bind close to the active site. A three-dimensional structure for human NAT1 has recently been released, and we have used this crystal structure to generate a model of the mouse Nat2 structure. We propose that a conformational change in the structure is required in order for ligands to bind to the active site of the protein.

## 1 Introduction

Arylamine *N*-acetyltransferases (NATs, EC 2.3.1.5) have traditionally been identified as drug-metabolising enzymes responsible for the metabolism of arylamines, arylhydroxylamines and arylhydrazines. NATs were first identified in man and a range of eukaryotes and have had an important part to play in the early years of identification of pharmacogenetic variation in response to drug treatment [1].

The hydrazine drug isoniazid is polymorphically *N*-acetylated in humans to its therapeutically inactive form by the human isoenzyme now known as human NAT2. There are two human NAT isoenzymes: human NAT2, which metabolises isoniazid, and human NAT1, which does not metabolise isoniazid or other arylhydrazines [2], but does catalyse the acetylation of a distinct but overlapping series of arylamines [2]. The pattern of expression of the two human *NAT* genes also differs. As a result of genome-wide microarray [3–12] and proteomic studies [13], it is clear that human NAT1 is highly expressed in oestrogen receptor positive breast cancer [14]. There is also evidence that the level of expression of human *NAT1* affects the growth of cultured breast cancer cells [13].

The role of the human NAT1 enzyme in breast cancer has not been extensively explored, although it has previously been demonstrated that the anti-oestrogen compound tamoxifen is an inhibitor of human NAT1 [15–17]. It has also been demonstrated that both human NAT1 and the oestrogen receptor are down-regulated in tissues in which p53 is mutated [5]. It appears that human NAT1 is a marker for oestrogen receptor positive breast cancer, although information is still accumulating on the relationship of this marker to others, including the oestrogen receptor itself [14]. However, if the human NAT1 enzyme is to be considered as a target for breast cancer therapy, it is essential that there is a suitable *in vivo* animal model for testing [18,19]. Mice represent the most convenient animal models of disease. At present, the status of mouse Nat2, the equivalent of human NAT1, in a model of breast cancer has not been explored. We have previously demonstrated that mouse *Nat2* is expressed in the epithelial cells lining the mammary ducts [20]. This is the same location as the human NAT1 enzyme in normal breast tissue [13,21].

In order to provide a firm foundation for establishing such a model, we have generated pure recombinant mouse Nat2 and investigated its activity with a wide range of substrates and the effects of a range of potential inhibitors, including endogenous and exogenous steroids.

## 2 Materials and methods

### 2.1 Chemicals

All chemicals were purchased from Sigma–Aldrich and all molecular biology reagents were purchased from Promega, unless otherwise stated.

### 2.2 Cloning and expression studies of mouse *Nat2*

The mouse *Nat2* open-reading frame [22] was sub-cloned into pET28b(+) (Novagen) using the compatible restriction enzyme sites, NdeI and EcoRI. This allowed the production of recombinant mouse NAT protein with an N-terminal His-tag, for ease of downstream purification. The pET28b(+) plasmid containing mouse *Nat2* was transformed into *Escherichia coli* strain JM109 and further transformed into Rosetta(DE3)pLysS (Novagen) after confirming the correct insert sequence (DNA Sequencing Facility, Biochemistry, University of Oxford). The positive transformant was grown in LB to mid-log phase at 37 °C with shaking (180 rpm) and stored at −80 °C with 10% (v/v) glycerol. Thawed glycerol stock (100 μL) was used to inoculate fresh LB media (100 mL) supplemented with kanamycin (30 μg/mL) and chloramphenicol (34 μg/mL) and the culture was incubated at 37 °C for 16 h with shaking (180 rpm). The starter culture was then diluted 50-fold into fresh LB media (2 L) supplemented with kanamycin (30 μg/mL), which was then incubated at 27 °C with shaking (180 rpm). When the absorbance at 600 nm reached 0.7, the expression of mouse Nat2 was induced by addition of isopropyl-*β*-d-thiogalactopyranoside (IPTG) to a final concentration of 0.1 mM. The culture was grown for a further 16 h and the cells were harvested by centrifugation (6000 × *g*, 4 °C, 20 min). The cell pellet was resuspended in 20 mL lysis buffer (300 mM NaCl, 20 mM Tris–HCl (pH 8.0) containing 1× EDTA-free Complete Protease Inhibitor (Roche)) and then stored at −80 °C.

For expression of ^15^N-labelled mouse Nat2, the starter culture was diluted 50-fold in ^15^N-labelled Standard-*E. coli*-OD2 N medium (Silantes) supplemented with kanamycin (30 μg/mL) and grown at 37 °C until the absorbance at 600 nm reached approximately 0.6. The cells were further grown at 27 °C until the absorbance reached 0.7, when the expression was induced with 0.1 mM IPTG. After induction for 17 h, the cells were harvested and stored as described for unlabelled mouse Nat2.

The resuspended frozen cells were thawed at 37 °C and sonicated on ice (25 cycles of 30 s on, 45 s off at 10 μm). The soluble lysate was isolated from the cell debris by centrifugation (12,000 × *g*, 4 °C, 20 min) and incubated (4 °C, 5 min) with 5 mL of Ni-NTA resin (Qiagen). The resin was sequentially washed with lysis buffer containing increasing imidazole concentrations in a step gradient at 4 °C (2 washes each of 10 mL of 0 mM, 10 mM, 20 mM, 50 mM and 100 mM imidazole). Fractions containing hexa-histidine tagged Nat2 (His-Nat2), were pooled and thrombin (5 U/mg NAT) was added to remove the His-tag. After 16 h incubation at 4 °C, the protein sample was dialysed against 20 mM Tris–HCl (pH 8.0), 1 mM dithiothreitol (DTT), 1 mM EDTA buffer, and concentrated to 1 mg/mL by centrifugation in a 15 mL concentrator (Amicon) after addition of 5% (v/v) glycerol. Protein samples in 100 μL aliquots were frozen in liquid nitrogen and stored at −80 °C.

Recombinant hamster Nat2 was expressed and purified as previously described [23]. ^15^N-labelled hamster Nat2 was grown and expressed as described for ^15^N-labelled mouse Nat2 and purified by the same method as for the unlabelled hamster Nat2.

The expression levels and activities of soluble Nat2 were monitored by visualizing the levels of recombinant protein expression on 12% acrylamide Tris–Glycine SDS-PAGE as well as by measuring the rate of acetylation of 4-aminobenzoic acid (PABA) as a substrate (see method below) [24].

### 2.3 Enzymic assays

#### 2.3.1 Acetylation of arylamines

The rates of arylamine acetylation by NATs were determined colorimetrically as previously described [25], but with minor modifications. Each assay contained the enzyme, arylamine substrate (see each figure legends for concentrations) and AcCoA (400 μM) in a total volume of 100 μL in assay buffer (20 mM Tris–HCl (pH 8.0), 1 mM DTT). Initially, the enzyme and substrate mixtures were pre-incubated at 25 °C for 5 min and AcCoA was added to start the reaction. The reaction was quenched using 20% (w/v) trichloroacetic acid (TCA) at different time intervals. The stopped reaction was centrifuged (16,000 × *g* rpm, 10 min) to pellet the precipitated proteins. The stopped reaction mixture (200 μL) was added to 800 μL of 5% (w/v) 4-(*N,N*-dimethylamino)benzaldehyde (DMAB) in 9:1 acetonitrile:water to develop the colour. The absorption at 450 nm was measured with a Hitachi U-2001 UV–vis spectrophotometer, with the amount of residual substrate in the reaction was determined by comparison with a standard curve.

#### 2.3.2 Hydrolysis of AcCoA (free CoA production)

The rate of production of the free thiol Coenzyme A by NAT, in the presence of a range of known NAT acetyl-acceptor substrates, was determined by using Ellman's reagent, 5,5′-dithio-bis(2-nitrobenzoic acid) (DTNB) as previously described [2,26]. The substrate (500 μM) and purified recombinant NAT were pre-incubated (25 °C, 5 min) in 96-well flat-bottomed polystyrene plates (Costar^®^, Corning Inc.) in 20 mM Tris–HCl (pH 8.0). AcCoA (400 μM) was added to start the reaction in a final volume of 100 μL. The reaction was quenched with the addition of 25 μL of guanidine–HCl solution (6.4 M guanidine–HCl, 0.1 M Tris–HCl (pH 7.3)) containing 5 mM DTNB. The absorbance at 405 nm was measured on a plate-reader (Sunrise, Tecan). Assay buffer was used to replace substrate, AcCoA or NAT for control reactions. The amount of CoA produced in the assay was determined in comparison with a CoA standard curve.

Substrate abbreviations used for the substrate selectivity assays are as follows: aniline (ANL), 4-aminobenzoic acid (PABA), 4-aminosalicylic acid (4AS), 5-aminosalicylic acid (5AS), 4-chloroaniline (CLA), 4-bromoaniline (BRA), 4-iodoaniline (IOA), 4-methoxyanline (ANS), 4-ethoxyaniline (EOA), 4-butoxyanline (BOA), 4-hexyloxyaniline (HOA), 4-phenoxyaniline (POA), 4-aminoveratrole (4AV), 2-aminofluorene (2AF), 4-aminobenzoyl-l-glutamate (pABGlu), sulfamethazine (SMZ), procainamide (PRO), 4-aminopyridine (APY), isoniazid (INH), hydralazine (HDZ) and phenylhydrazine (PHZ). The stock concentration of substrate was 100 mM dissolved in DMSO, such that the final concentration of DMSO was less than 5% in the assays. DMSO only controls were carried out.

### 2.4 NMR spectroscopy

The 2D ^1^H-^15^N HSQC spectrum of mouse Nat2 was collected using a 750 MHz NMR spectrometer (Department of Biochemistry, University of Oxford). The spectrum was collected at 20 °C for a sample containing 30 mg/mL of uniformly ^15^N-labelled mouse Nat2 in 95% H_2_O/5% D_2_O 10 mM Tris buffer at pH 7.0. Spectral widths of 12,500 and 2500 Hz were used in F_2_ and F_1_, respectively. The dataset contained 1024 and 128 complex points in *t*_2_ and *t*_1_, respectively.

1D ^1^H NMR spectra for hamster and mouse Nat2 were collected at 20 °C using a jump-return sequence. This sequence enabled the observation of H^N^ peaks which exchange to a significant extent with H_2_O. Spectra were collected using a 600 MHz spectrometer using a sweep width of 12,500 Hz. Unlabelled hamster and mouse Nat2 were used at a concentration of 10 mg/mL in the buffer described above. The titration of mouse Nat2 with bisphenol A was carried out by stepwise addition of small volumes (5–20 μL) of a concentrated solution of bisphenol A (100 mM in deuterated DMSO) to the mouse Nat2 solution in the NMR tube. The highest concentration of DMSO in the sample used was 2.8%. DMSO alone had no effect on the NMR spectrum of mouse Nat2 using concentrations up to 5%.

## 3 Results

### 3.1 Cloning and expression of mouse *Nat2*

**Fig. 1:**
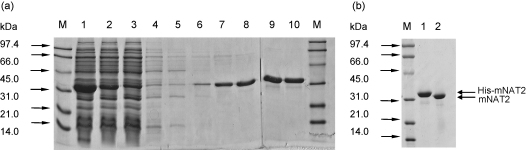
Heterologous expression and purification of recombinant mouse Nat2 in *E. coli*. SDS-PAGE analysis of expression and affinity purification of mouse Nat2. Mouse Nat2 was produced in *E. coli* Rosetta(DE3)pLysS strain and purified by Ni-NTA affinity chromatography. The purification gel shows expression of His-Nat2 at 34 kDa. Lanes: 1, whole cells; 2, soluble fraction; 3, unbound wash; 4, 0 mM imidazole (IMZ) wash; 5, 1 mM IMZ wash; 6, 10 mM IMZ wash; 7, 20 mM IMZ wash; 8, 50 mM IMZ wash; 9, first 100 mM IMZ wash; 10, second 100 mM IMZ wash; M, low-range molecular weight markers (BioRad). Thrombin cleavage of His-Nat2. Purified His-Nat2 was incubated with thrombin (5 U/mg NAT) at 4 °C for 6 h for complete His-tag cleavage. Lanes: M, low-range molecular weight marker (BioRad); 1, His-Nat2 only (8 μg); 2, thrombin-treated, Nat2 (8 μg).

The mouse *Nat2* was cloned into *E. coli* expression vector pET28b(+) and expressed in *E. coli* Rosetta (DE3)pLysS with an N-terminal hexa-histidine tag. The recombinant mouse His-Nat2 was purified using immobilised metal affinity chromatography, using a Ni-NTA column (Novagen), and eluted (Fig. 1a). Pure Nat2 predominantly eluted in the 50 mM and 100 mM imidazole washes. The hexa-histidine tag was readily removed by thrombin digestion (Fig. 1b). The purified recombinant Nat2 was active, readily catalysing the acetylation of PABA, a well-studied arylamine substrate of mouse Nat2 [27] (Table 1). The enzyme was relatively stable, and the enzymic activity was maintained (>85%) after incubation at temperatures ranging between 4 °C and 25 °C over a 72 h period.

### 3.2 Substrate specificity of mouse Nat2

To further characterise the purified recombinant mouse Nat2, a panel of known NAT substrates (20 compounds) was tested to determine the substrate selectivity of recombinant mouse Nat2. In order to allow direct comparison with substrate selectivity profile for human NAT1 and NAT2, the assay for mouse Nat2 was performed under the same conditions as previously described [2].

**Fig. 2:**
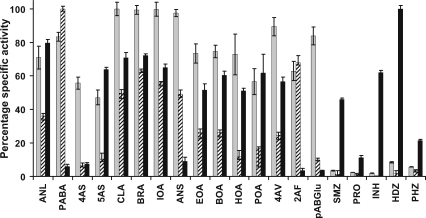
Comparison of the substrate specific activity profiles of mouse Nat2, human NAT1 and human NAT2. The substrate specific activity profiles for mouse Nat2 (grey bars), human NAT1 (cross-hatched bars) and human NAT2 (black bars) are shown. The activity of mouse Nat2 was determined by measuring the rate of CoA production, as described in Section 2. Purified mouse Nat2 (0.125 μg) was incubated with arylamine substrate (each at 500 μM) and AcCoA (400 μM) in assay buffer (20 mM Tris–HCl (pH 8.0)) containing 0.5% (v/v) DMSO at 25 °C. The specific activities were determined from the linear initial rates of reaction. All measurements were performed in triplicate and are expressed as mean ± standard deviation relative to the most rapidly acetylated substrate. The human NAT1 and NAT2 data are taken from [2]. The abbreviations are defined in Section 2.

Mouse Nat2 shows high specific activities with a broad range of arylamines, including PABA, 5AS, 4AS and 2AF (Fig. 2 and Supplementary Table 1). In contrast, mouse Nat2 shows low specific activities against arylhydrazine substrates such as HDZ, INH, and PHZ, and against arylamine drugs procainamide and sulfamethazine (Fig. 2). This overall trend is in agreement with the substrate selectivity observed in previous studies with impure recombinant mouse Nat2 using a subset of these compounds [22,27,28], although it is much less than the activity observed towards these substrates with human NAT2 (Fig. 2). There was low, yet measurable activity using arylhydrazine substrates with purified mouse Nat2, which has not been observed in previous studies using recombinant mouse Nat2 expressed in a transient expression system [28]. Pure recombinant mouse Nat2 also readily acetylates 2-aminofluorene, the substrate which has been used to identify mouse strains carrying an active form of mouse Nat2—the fast acetylating C57Bl/6 strain, rather than the inactive form as is found in the slow acetylating A/J strain which is unstable and likely to be subject to rapid degradation [29].

The specific activity pattern of human NAT1 [2] overlaps with the mouse Nat2 profile (Fig. 2), reconfirming that mouse Nat2 is very similar to human NAT1 in substrate specificity. Mouse Nat2 shows differences from the activity profile of human NAT1, however. It appears that mouse Nat2 generally has higher inherent specific activity than human NAT1 and also acetylates arylhydrazines, when the acetylation of these compounds by human NAT1 is not detected under the same conditions [2].

### 3.3 Mouse Nat2 inhibition by steroidogenic compounds

It had been demonstrated, with extracts of tissues and cells now known to express the human *NAT1* gene, that tamoxifen is an inhibitor of human NAT1 [15–17]. In view of the relationship between the expression of human NAT1 and oestrogen receptor positivity in breast cancer [14], we wished to investigate the effects of steroidogenic compounds and xenobiotic oestrogenic compounds, including tamoxifen, on the activity of mouse Nat2.

From the inhibition study using a broad range of steroidogenic compounds, it has been demonstrated that mouse Nat2 is inhibited selectively by oestrogenic compounds, with 17-hydroxy-β-estradiol identified as the most potent inhibitor (Table 2 and Supplementary Fig. 1). This was substantiated by the inhibition of mouse Nat2 activity by xenobiotic oestrogens, such as tamoxifen, and the IC_50_ values for these inhibitors are shown in Table 3. The phytoestrogens genistein, alpha-zearalenol and the synthetic oestrogen diethylstilbestrol show similar potency of mouse Nat2 inhibition to 17-hydroxy-*β*-estradiol, tamoxifen and 4-hydroxy tamoxifen. Bisphenol A was also shown to be an inhibitor of mouse Nat2, albeit with lower potency than some of the other compounds tested. One key practical advantage of using bisphenol A as an inhibitor is the improved solubility of this compound relative to the other phytoestrogens and steroids.

### 3.4 Structure of mouse Nat2

**Fig. 3:**
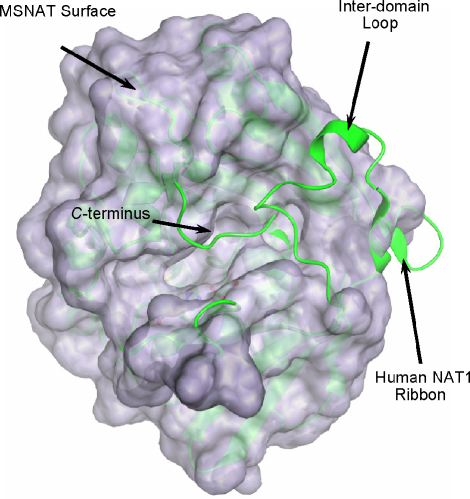
Comparison of the crystal structures of NAT from *M. smegmatis* (MSNAT) and human NAT1. The surface shown was calculated from the MSNAT crystal structure (PDB accession code 1GX3), and the human NAT1 crystal structure F125S mutant (PDB accession code 2IJA) is shown in ribbon format. The C*-*terminus and inter-domain loop region of human NAT1 protrude from the eukaryotic protein core, and both of these protein regions block the prokaryotic NAT active site. The C-terminus of human NAT1 is found in the region termed the ‘β-site’ [47].

Until recently, there has been no structural information on eukaryotic NAT enzymes. However, recently the three-dimensional structure of a human NAT1 mutant (Phe/Ser substitution at position 125) was determined by X-ray crystallography (PDB accession code: 2IJA [30]). A non-mutated human NAT1 crystal structure has also been deposited in which the active-site cysteine residue was modified to *S*-(2-anilino-2-oxoethyl)-cysteine (PDB accession code: 2PQT). It is considered likely that both the mutation of residue 125 and the modification of the active-site cysteine residue stabilise the structure of the human enzyme, because thousands of attempts to generate a crystal from the native human NAT1 protein have been unsuccessful (A. Kawamura, unpublished results). The structure of human NAT1 is illustrated in comparison with the NAT from *M. smegmatis* (Fig. 3). The eukaryotic proteins contain a loop between the second and third domains. This inter-domain loop is not present in their prokaryotic counterparts [31], as highlighted in Fig. 3. It is interesting to note that in the structure of the human NAT1 enzyme, this inter-domain loop is folded back over the active site. Likewise, the C-terminus is shown to be folded over the active-site cleft. These observations indicate that a conformational change in the protein may be required to allow the substrates to access the active-site cysteine residue. However, a structure of human NAT2 with coenzyme A bound has also been deposited (PDB accession code: 2PFR), and the position of the coenzyme A is such that the C-terminus and inter-domain loops regions are not significantly changed. This is in contrast to the recently reported structure of *Mycobacterium marinum* NAT in complex with coenzyme A [32]. In this bacterial NAT, the C-terminus is shorter than in the eukaryotic structures, and there is no inter-domain loop, and the ligand is found in the space where these regions are located in the eukaryotic structure.

**Fig. 4:**
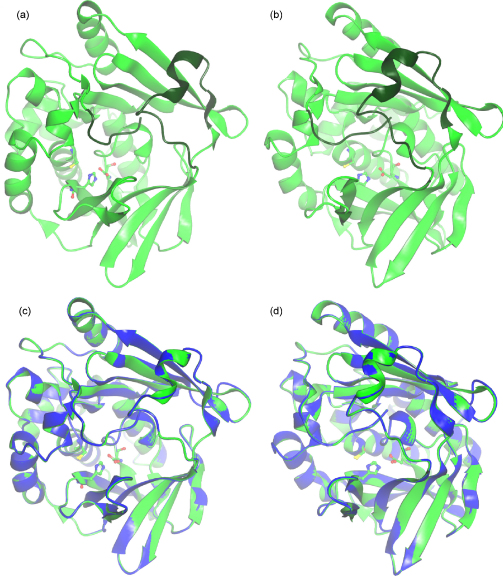
(a) and (b) The structure of mouse Nat2. The mouse Nat2 protein structure was produced by homology modelling with the program Modeller 8v2 [33]. The human NAT1 crystal structure (PDB code 2IJA) was used as a template, and the amino acid sequences of human NAT1 and mouse Nat2 were aligned with the program ClustalW [48]. The mouse Nat2 ribbon structure is shown in green. The ribbon structures of the inter-domain loop region and C-terminal hexapeptide residues (residues 168–184 and 285–290) are shown in dark green, and the active site catalytic triad (Cys^68^, His^107^ and Asp^122^) residues are shown in ball and stick representation. The pdb file of the mouse Nat2 homology model is available upon request. The figure was produced with Aesop, as previously described [49]. (c) and (d) A comparison of the human NAT1 crystal structure (PDB code 2IJA) and the mouse Nat2 homology model. The human NAT1 crystal structure is shown in blue and the mouse Nat2 model is shown in green. The two proteins share 82% identity at the amino acid level. Over 1191 equivalent atoms, the two structures have a root mean squared deviation of 0.75 Å. The catalytic triad residues for both proteins are shown in ball and stick representation. The views in (b) and (d) were obtained by rotating the structures shown in (a) and (c) by 30°. (For interpretation of the references to colour in this figure legend, the reader is referred to the web version of the article.)

We have generated a structural model of the mouse Nat2 protein, whose amino acid sequence is over 80% identical to human NAT1, by using the program Modeller 8v2 [33]. Fig. 4 shows the structural model of mouse Nat2 and a comparison of the mouse Nat2 and human NAT1 protein structures. The two structures share 1191 equivalent atoms, over which the root mean squared deviation is 0.75 Å. In mouse Nat2, as in human NAT1, the inter-domain loop and the C-terminus occlude the active site. Despite the evidence for coenzyme A binding to human NAT2 without major conformational rearrangement of the C-terminus and inter-domain loop regions, it has not been possible to perform ligand docking into the mouse Nat2 or human NAT1 structures without disturbing these regions of the protein structure. Therefore, it remains possible that the inter-domain loop regions and C-termini of the eukaryotic NAT proteins are conformationally flexible, in order to accommodate substrate or inhibitor binding.

### 3.5 NMR studies

**Fig. 5:**
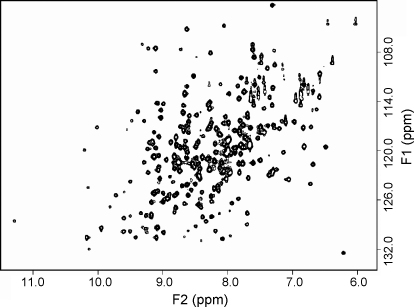
750 MHz 2D ^1^H-^15^N HSQC spectrum of mouse Nat2.

NMR studies are ideal for investigating protein interactions with substrates and inhibitors in solution. Therefore, we have generated mouse Nat2 uniformly labelled with ^15^N to allow 2D NMR investigations of the protein. The 2D ^1^H-^15^N HSQC spectrum of mouse Nat2 is shown in Fig. 5; this spectrum contains a peak for each backbone amide (^1^H^N^-^15^N) and additional peaks from the side chains of Asn, Gln and Trp. The HSQC spectrum is well resolved, as expected for a compact globular structure, and some cross peaks are relatively sharp for a 30 kDa protein, this may indicate some flexibility between the domains of mouse Nat2 or the presence of mobile loops. The HSQC spectrum of mouse Nat2 has been compared with the corresponding spectrum from hamster Nat2 for which a full assignment is available [34]. The pattern of peaks in the HSQC spectra is extremely similar for the two homologous proteins; this allows some peaks observed in the mouse Nat2 spectrum to be assigned on the basis of the hamster Nat2 spectrum.

**Fig. 6:**
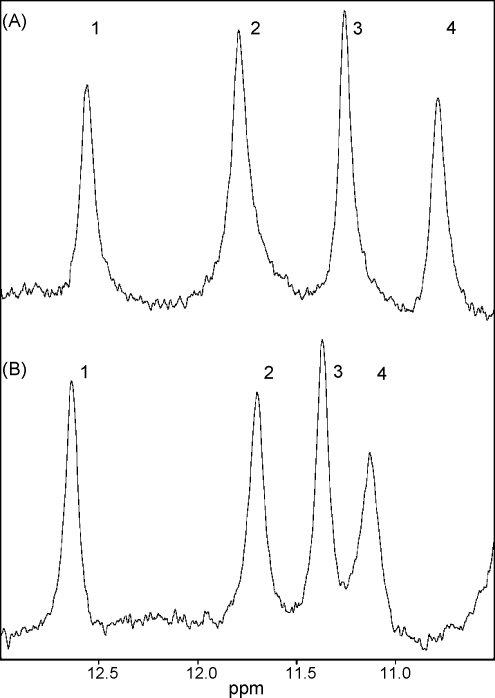
The downfield region of the 1D ^1^H NMR spectra of (A) mouse and (B) hamster Nat2 collected using a jump-return pulse sequence. These peaks arise from the indole H^N^ of two tryptophan residues (peaks 2 and 3, Trp^67^ and Trp^132^ respectively) and from imidazole H^N^ of histidine (peaks 1 and 4, His).

**Fig. 7:**
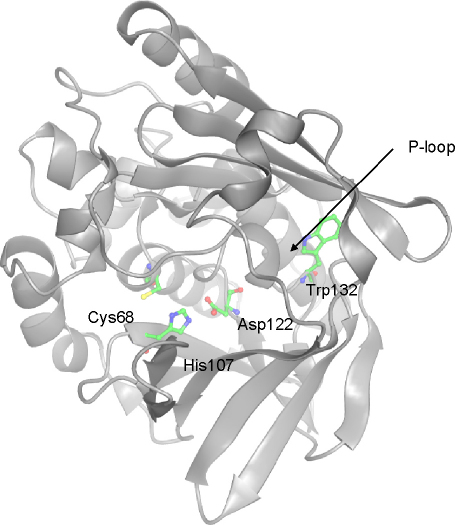
The mouse Nat2 homology model, based on the crystal structure of human NAT1, showing the position of Trp^132^ relative to the active-site triad (shown in ball and stick representation). The putative phosphate-binding P-loop is shown.

The downfield region of the ^1^H 1D spectra, collected with a jump-return sequence, of mouse and hamster Nat2 also show a similar pattern of peaks (Fig. 6A and B). The four peaks are compared in Table 4, together with their assignments. Two of the peaks (2 and 3) have been assigned previously for hamster Nat2 and can be assigned in the mouse spectrum by homology; these correspond to strongly hydrogen bonded tryptophan indole groups: the tryptophan associated with the P-loop (Trp^132^, see Fig. 7) and Trp^67^, which is located adjacent to the active-site Cys^68^, and appears to have a structural role. The strong hydrogen bonds are likely to be responsible for the large downfield shifts of these tryptophan peaks compared to random coil values (∼9.5 ppm). The two remaining peaks (1 and 4) are assigned to the imidazole ^1^H^N^ of histidine on the basis of ^15^N decoupling experiments; these peaks have not been assigned previously for hamster Nat2. The side chain ^1^H^N^ groups of histidine are usually not observed by NMR, particularly at pH 7, due to their rapid exchange with solvent protons. The observation of these peaks indicates that the histidine residues are probably buried within the protein and that the side chain ^1^H^N^ are involved in hydrogen bonds. Analysis of the homology model of mouse Nat2 suggests that at least one of these peaks might arise from the active site histidine, His^107^. The homology model of mouse Nat 2 indicates that the side chain of His^107^ is involved in two hydrogen bonds in the active site and is the least accessible to solvent. In addition, recent enzymological studies with hamster Nat2 have confirmed that the active-site cysteine and histidine residues exist as a thiolate-imidazolium ion pair [35], in which the histidine has two tightly bound and H-bonded protons.

### 3.6 Interaction of mouse Nat2 with exogenous steroid inhibitors

**Fig. 8:**
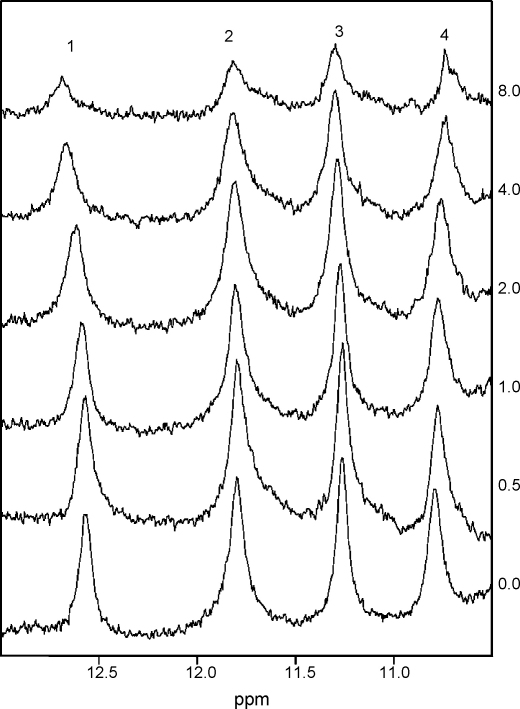
The downfield region of the 1D ^1^H NMR spectra obtained in a titration of mouse Nat2 with bisphenol A. Spectra collected with 0–8 equivalents of bisphenol A are shown. Peaks are observed to shift and to broaden as increasing amounts of bisphenol A are added; this broadening may indicate chemical exchange within the bound protein. Some precipitation of protein was observed with 8 equivalents of bisphenol A, which is likely to account for the decrease in peak intensity in this spectrum.

**Fig. 9:**
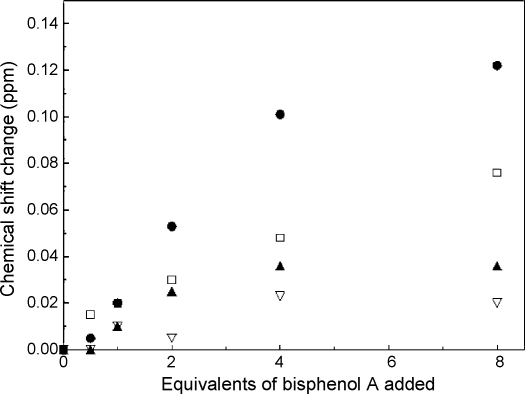
Titration of mouse Nat2 with bisphenol A. The magnitudes of changes in chemical shift observed for the four downfield shifted peaks are shown as a function of the amount of added bisphenol A. The largest shifts are observed for peaks 1 (filled circle) and 4 (open square) which arise from histidine; smaller shifts are observed for peaks 2 (open triangle) and 3 (filled triangle).

In order to probe the molecular interactions of mouse Nat2 and the exogenous steroid inhibitors, we have investigated the effect of bisphenol A on the 1D ^1^H NMR spectrum of mouse Nat2 (Figs. 8 and 9). The observation of changes in chemical shift for particular amino acid residues which result from the addition of a ligand are usually interpreted as an indication that these residues are located in close proximity to the ligand-binding site. When bisphenol A was titrated into a solution of mouse Nat2, the two downfield histidine peaks (1 and 4, Table 4) were observed to shift by more than 0.05 ppm (Fig. 9); peak 1 shifts downfield by ∼0.12 ppm and peak 4 shifts upfield by ∼0.08 ppm in the presence of 8 equivalents of bisphenol A. Additional small shifts were also observed for the side chain H^N^ of Trp^67^ and Trp^132^ (Fig. 9). The observation of progressive changes in chemical shift upon addition of bisphenol A indicates fast exchange on the NMR timescale; this would be expected given the relatively weak affinity of mouse Nat2 for this ligand (IC_50_ = 290 ± 10 μM). We have postulated above that at least one of the histidine peaks arises from the active-site histidine, His^107^; therefore, these results show that binding of bisphenol A is likely to take place in close proximity to the active site of mouse Nat2.

## 4 Discussion

The possibility that human NAT1 may serve as a marker for sub-dividing populations of different breast cancers is intriguing. It may also be that human NAT1 will find a role as a target for breast cancer therapies. In order to exploit these findings fully, it will be essential to have an animal model. There have been, for a long time, suggestions that human NAT1 has an endogenous role. The mouse equivalent of human NAT1 is mouse Nat2. Genetic deletion of mouse *Nat2* has not created an overt phenotype; however, some of these studies in mice indicate that *Nat2* deletion gives rise to skewed sex ratios both on mixed [36] and pure [37] genetic backgrounds, indicating a gender-dependent phenotype, discernable at the population level, that is consistent with a role in endocrine function. There is now also clear evidence that mouse Nat2 has a role *in vivo* in folate catabolism [38]. These studies, together with the data on human NAT1 and breast cancer, are intriguing and are likely to provide clues as to the endogenous role of NAT1. The data presented increases the knowledge of mouse Nat2 in comparison with human NAT1 and reinforce the notion that mouse Nat2 is a good homologue of human NAT1. Mouse Nat2 has a similar substrate specific activity profile to human NAT1.

The inhibition studies show that mouse Nat2 is inhibited by endogenous steroids and, like human NAT1, is inhibited by tamoxifen. Bisphenol A, which is used for making polycarbonates [39], has been reported to activate the oestrogen receptor [40]. Like tamoxifen, it is also an inhibitor of mouse Nat2. We have used bisphenol A to look at the binding of an oestrogenic stimulatory compound to mouse Nat2 and show by NMR that bisphenol A interacts with mouse Nat2 close to the active site region. These studies could not be done directly with tamoxifen because of the high concentrations of protein (and hence ligand) required for NMR experiments under which conditions tamoxifen is insoluble. Both endogenous and xenobiotic oestrogens have been shown to modify breast cancer risk, and tamoxifen is widely used for breast cancer therapy (reviewed in [41]). The effect of different synthetic or natural oestrogens on cell proliferation has been analysed directly, in breast cancer cell lines, and in gene expression studies [41–44]. Although the dose–response curves for different oestrogenic compounds vary with the assay system, bisphenol A is generally less active than tamoxifen, genistein or diethylstilbestrol, which reflects the order of potency of mouse Nat2 inhibition. Circulating plasma concentrations of the phytoestogen genistein have been found to be as high as 18 μM after a soy-based meal [45], and intratumoral levels of tamoxifen can reach micromolar concentrations [46]. Whether the effects on inhibition of Nat2 activity in vitro may be physiologically relevant for breast cancer prevention or therapy will require further analysis.

These studies pave the way for investigation of the role of mouse Nat2, the human NAT1 homologue, in studies of oestrogen receptor positive breast cancer. This is particularly relevant in view of the observation that mouse Nat2 is located histologically in the mammary gland epithelial cells [20]. The structural studies also provide a rational basis for identification of mouse Nat2 inhibitors. We have recently screened a 5000-strong compound library using human NAT1, human NAT2, mouse Nat1 and mouse Nat2; from these studies, several compounds been have identified which are potent and specific inhibitors of human NAT1 and its mouse homologue, Nat2. An accumulation of information on the eukaryotic NAT proteins at a structural level will allow further development of chemical tools for investigation of the role of this marker in breast cancer in humans, and its potential in animal models.

## Figures and Tables

**Table 1:** Purification of mouse Nat2

	Volume (mL)	Protein concentration (mg/mL)	Total protein (mg)	Specific activity (μmol/min/mg)	Total activity (μmol/min)	Percentage recovery
Culture	1000					
Cell lysate*	15	35	531	5	2390	
NAT containing IMZ wash (50–100 mM)**	25	2	58	29	1682	70
Thrombin cleaved NAT**	25	1.9	47.5	28	1330	56

NAT activities were measured by determining the rate of acetylation of the arylamine substrate, PABA (100 μM), as described in Section 2. Protein concentrations were measured by using the Bradford assay (*) and absorbance (**) at 280 nm (ɛ280_mNAT2_ = 40920 M^−1^ cm^−1^).

**Table 2:** NAT inhibition by steroidogenic compounds

	IC_50_ (μM)
Pregnelone	>100
17-Hydroxy-progesterone	>500
Androsteinedione	>500
Testosterone	>500
Estrone	370 ± 10
17-Hydroxy-β-estradiol	55 ± 5

The steroidogenic compounds which have shown inhibition against mouse Nat2 (Supplementary Fig. 1) were further investigated and their IC_50_ values were determined by varying the inhibitor concentrations (1–500 μM) under the same experimental conditions as described in Supplementary Fig. 1.

**Table 3:** Mouse Nat2 inhibitory concentration (IC_50_) of xenobiotic oestrogens

Xenobiotic estrogens	Structures	IC_50_ mNAT2 (μM)
Tamoxifen		57 ± 3
4-OH tamoxifen		65 ± 15
Diethylstilbestrol (E)-3,4-Bis(4-hydroxyphenyl)-3-hexene		∼50
Zearalenone		220 ± 10
Alpha-Zearalenol		70 ± 10
Beta-Zearalenol		450 ± 10
Bisphenol A		290 ± 10
Geni stein		45 ± 5

Half-maximal inhibitory concentrations of xenobiotic oestrogens against mouse Nat2 were measured as previously described (Supplementary Fig. 1).

**Table 4:** Summary of peaks observed in the downfield region (10.5–13 ppm) of the spectra of hamster and mouse Nat2

Peak	Hamster Nat2 ^1^H δ (ppm)	Mouse Nat2 ^1^H δ (ppm)	Mouse Nat2 ^15^N δ (ppm)	Assignment
1	12.64	12.57	170.2	Histidine side chain H^N^
2	11.70	11.80	132.0	Trp^67^ indole H^N^
3	11.38	11.26	128.6	Trp^132^ indole H^N^
4	11.13	10.79	165.2	Histidine side chain H^N^

## Appendix A Supplementary data

Supplementary Fig. 1 Inhibition of purified recombinant Nat2 by steroids. The inhibitory effect of steroids on the rate of acetylation of arylamines by mouse Nat2 was measured by using the colorimetric DMAB assay as described in Section 2. Test compounds (50 μM, 2% DMSO unless specified) were pre-incubated with recombinant mouse Nat2 (3 ng) and PABA (75 μM) for 10 min at room temperature. AcCoA (400 μM) was added to start the reaction and the reaction mixture was incubated at 37 °C. The total reaction volume was 100 μL. Reactions were stopped at various time intervals by the addition of 100 μL of 20% (w/v) TCA. The colour was developed and measured as described in Section 2. The linear initial rate of reaction was determined and the percentage inhibition was calculated relative to the control activity measured without the inhibitor. All measurements were performed in triplicate and are expressed as mean ± standard deviation. *2% (v/v) ethanol **5% (v/v) DMSO used to dissolve the compounds in the assay.
