# Supplementary figures and images for: Mouse N-acetyltransferase type 2, the homologue of human N-acetyltransferase type 1
Source: Biochem Pharmacol. 2008 Apr 1;75(7):1550–60. doi: 10.1016/j.bcp.2007.12.012 (PMC2279149; doi:10.1016/j.bcp.2007.12.012)

## Slide 1
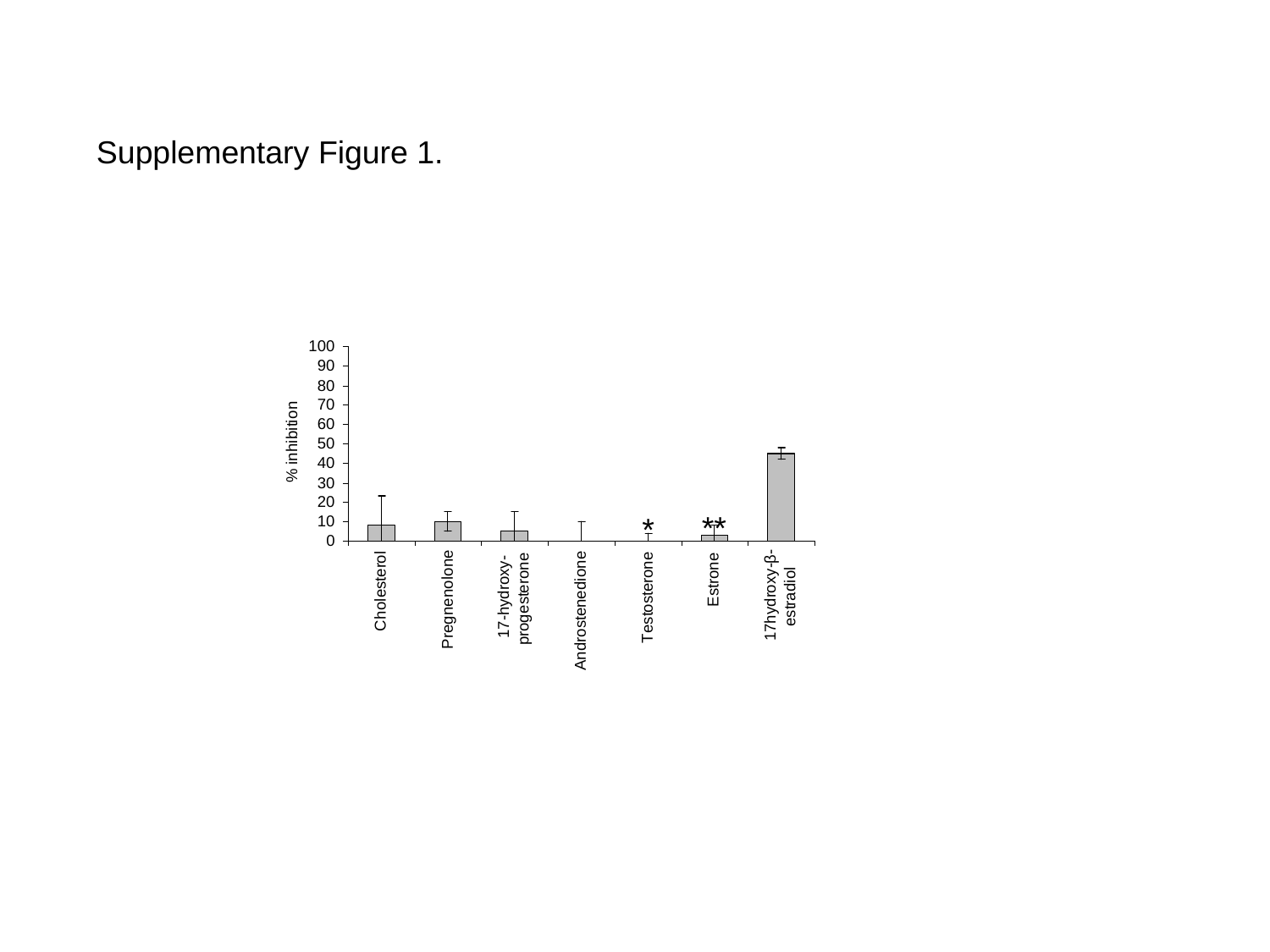

Supplementary Figure 1.
**
*

Supplement: Supplementary file 1 [file mmc1.ppt]
