# Supplementary material for: Mouse N-acetyltransferase type 2, the homologue of human N-acetyltransferase type 1
Source: Biochem Pharmacol. 2008 Apr 1;75(7):1550–60. doi: 10.1016/j.bcp.2007.12.012 (PMC2279149; doi:10.1016/j.bcp.2007.12.012)
Supplement: Supplementary file 2 [file mmc2.doc]

**Supplementary Table 1.**

**Specific activities of mouse Nat2 against known substrates of NATs.**

The rate of hydrolysis of AcCoA was measured in the presence of a wide range of acetyl-acceptors (known NAT substrates, at 500 μM) and mouse Nat2 as described in the Methods section and Supplementary Fig. 1. The assays were performed in the presence of 5% (*v/v*) DMSO.
